# Effects of Social Structure on Effective Population Size Change Estimates

**DOI:** 10.1111/eva.70063

**Published:** 2025-01-14

**Authors:** Bárbara Ribeiro Parreira, Shyam Gopalakrishnan, Lounès Chikhi

**Affiliations:** ^1^ Center for Evolutionary Hologenomics Globe Institute, University of Copenhagen Copenhagen Denmark; ^2^ Instituto Gulbenkian de Ciência Oeiras Portugal; ^3^ Centre de Recherche sur la Biodiversité et l'Environnement (CRBE) UMR 5300 Université de Toulouse, CNRS, IRD, Toulouse INP, Université Toulouse 3 Paul Sabatier (UT3) Toulouse France; ^4^ Centre for Ecology, Evolution and Environmental Changes (cE3c) Faculdade de Ciências da Universidade de Lisboa Lisboa Portugal

**Keywords:** demographic inference, effective size, population structure, social structure

## Abstract

Most methods currently used to infer the “demographic history of species” interpret this expression as a history of population size changes. The detection, quantification, and dating of demographic changes often rely on the assumption that population structure can be neglected. However, most vertebrates are typically organized in populations subdivided into social groups that are usually ignored in the interpretation of genetic data. This could be problematic since an increasing number of studies have shown that population structure can generate spurious signatures of population size change. Here, we simulate microsatellite data from a species subdivided into social groups where reproduction occurs according to different mating systems (monogamy, polygynandry, and polygyny). We estimate the effective population size (*N*_e_) and quantify the effect of social structure on estimates of changes in *N*_e_. We analyze the simulated data with two widely used methods for demographic inference. The first approach, BOTTLENECK, tests whether the samples are at mutation–drift equilibrium and thus whether a single *N*_e_ can be estimated. The second approach, msvar, aims at quantifying and dating changes in *N*_e_. We find that social structure may lead to signals of departure from mutation–drift equilibrium including signals of expansion and bottlenecks. We also find that expansion signals may be observed under simple stationary Wright–Fisher models with low diversity. Since small populations tend to characterize many endangered species, we stress that methods trying to infer *N*_e_ should be interpreted with care and validated with simulated data incorporating information about structure. Spurious expansion signals due to social structure can mask critical population size changes. These can obscure true bottleneck events and be particularly problematic in endangered species.

## 1 Introduction

Conservation genetics aims at using genetic data to improve conservation decisions. It typically requires the sampling of natural populations from which genetic, and now genomic data are obtained and interpreted (Oklander and Soto‐Calderón 2024). A first step consists in producing metrics such as the number of alleles and the observed and expected heterozygosities, and interpreting them using concepts from population genetics. One key concept is the effective population size, *N*_e_, introduced by Wright (1931) as a way to summarize the genetic properties of populations. The effective size is the size of an ideal population that would have the same properties, say inbreeding, as the real population of interest (see review by Charlesworth 2009). The effective size is an important concept because it provides a metric that allows comparisons between species that often differ in many biological characteristics such as the sex ratio, the variance in reproductive success, or its demographic history.

The demographic history of populations is often interpreted as the history of change in *N*_e_ over time (Beaumont 1999; Li and Durbin 2011; Liu and Fu 2020; Novo et al. 2023). While some demographic inference methods focus on the recent past, including the past few thousands of years (Beaumont 1999; Liu and Fu 2020; Novo et al. 2023), others focus on the deeper past (Li and Durbin 2011). Some methods estimate a single contemporary *N*_e_, supposedly reflecting the current *N*_e_, and others estimate a limited number of past effective sizes. For instance, the heterozygosity excess test relies on the equilibrium properties of summary statistics and determines whether a single *N*_e_ can explain the observed statistics. This method detects departures from Wright–Fisher expectations (Ewens 1972; Nei, Maruyama, and Chakraborty 1975; Tajima 1989). Such departures from equilibrium can be difficult to interpret because the properties of genetic data (coalescence rates, loss of heterozygosity over time, etc.) are influenced by non‐panmitic conditions. Inferred changes in *N*_e_ may thus reflect other properties of the populations or species of interest, such as population structure or changes in migration. Whichever method one uses, the relationship between the inferred *N*_e_ and the actual demography of the species, such as its census size (*N*_c_), remains complex. In the conservation genetics context, it is crucial to clarify what genetic data allow to say, particularly when we compute numbers and estimate parameters that are as central as *N*_e_ (Charlesworth 2009; Sjödin et al. 2005), as this Special Issue stresses.

Many demographic inference methods assume that samples have been taken from panmictic and isolated populations, hence assuming that population connectivity is negligible. However, population structure plays a crucial role in generating patterns of genetic diversity and differentiation. Its importance has long been recognized in both field and theoretical studies. A number of methods and simulation programs have been developed to either infer demographic parameters (see Arredondo et al. 2021; Beerli and Felsenstein 2001; Chikhi, Bruford, and Beaumont 2001; Hey and Nielsen 2004; Wang et al. 2020), or to simulate genetic data under complex structured models (Excoffier, Estoup, and Cornuet 2005; Excoffier et al. 2021; Hudson 2002). A growing number of studies have found that population structure can generate spurious signals of demographic change. These studies have shown that ignoring population structure may lead to the detection of apparent size changes in populations for which such changes never took place. For instance, apparent changes in *N*_e_ may be due to structure or fluctuations in connectivity (Beaumont 2003; Chikhi et al. 2018; Girod et al. 2011; Novo et al. 2023; Paz‐Vinas et al. 2013; Wakeley 1999). Research in this area has also questioned the very notion of *N*_e_. Under some structured models, *N*_e_ cannot be defined or can depend on the sampling scheme, making it a misleading concept (Chikhi et al. 2018; Sjödin et al. 2005). In addition, the sampling scheme can either contribute to (or minimize) the detection of spurious size changes. For example, spurious changes may be minimized when several individuals are collected from different demes rather than from one single deme (Chikhi et al. 2010; Wakeley 1999). Moreover, for many species we usually have limited information on population structure before sampling and analyzing the data. Thus, the effects of the sampling scheme and of population structure are strongly connected and difficult to separate.

So far, studies have looked at classical population genetics models of structure, such as *n*‐island, stepping‐stone, tree and continent‐island models, which assume demes (random‐mating units) as the fundamental unit of population structure. But, real populations are rarely structured in clearly identifiable demes. They are structured in a complex variety of ways including age classes, sex ratio, and mating systems, which may create deviations from the standard coalescent (Wakeley 2009). Firstly, deviations from random mating occur because age and social structure can prevent some individuals from participating in reproduction. In most mammalian societies a few (dominant) males try to control access to females, and in some species, a small number of females can monopolize reproduction—in extreme cases one single female breeds while others do not reproduce at all during their entire lifespan (Clutton‐Brock 2006, 2009). Secondly, dispersal can be strongly sex‐biased, with one sex dispersing away from the natal group before reproduction and the other being philopatric (Greenwood 1980; Johnson and Gaines 1990), and this too may generate different gene genealogies compared to stationary random mating populations.

Moreover, studies based on unlinked single nucleotide polymorphisms (SNPs) have shown that dioecious reproduction, high reproductive skew, and inbreeding constraints (mating prevented or preferred among sibs) can affect the expected time to the common ancestor and therefore the inferred coalescent *N*_e_ (Campbell 2015). In addition, social structure may influence statistical associations across loci, or other statistics that are increasingly used to estimate contemporary *N*_e_ (Novo et al. 2023). However, all these results have not yet been integrated into demographic inference. Little is known about the consequence of applying classical inferential methods to social species and in particular, whether these consequences differ from those expected under standard population genetics models.

Here, we simulate microsatellite data under socially structured populations. Even though genome‐wide SNPs are now increasingly used for non‐model species, microsatellites are still among the markers most used in conservation genetics. Microsatellites are extensively used for determining genetic diversity and demographic history in many threatened populations (Ghazi et al. 2021; Modi et al. 2021; Srinivas and Jhala 2024). Moreover, for many rare and elusive species, non‐invasive sampling makes it difficult to take advantage of whole genome data and many studies continue to focus on microsatellites due to low endogenous content and highly fragmented DNA (Bajwa et al. 2023). We analyze simulated data using two approaches as implemented in the BOTTLENECK (Piry, Luikart, and Cornuet 1999) and the msvar (Beaumont 1999) softwares. We also estimate contemporary *N*_e_ using an approach based on linkage disequilibrium patterns between unlinked loci (Do et al. 2014). Our aim is to understand the extent to which properties of socially structured populations, such as dioecious reproduction, complex mating systems, and age‐classes bias the estimation of recent *N*_e_ and *N*_e_ changes.

## 2 Materials and Methods

### 2.1 Generating Data Under Socially Structured Populations

We simulated genetic data under a socially structured population. We used an individual‐based forward‐in‐time simulation framework developed and formalized by Parreira and Chikhi (2015), where the model is described in detail. In short, the model assumes that a population is a fully connected network of social groups among which individuals can disperse. Under this topology each social group is connected to any other in the population. There is structure but no space, as in Wright's *n*‐island model (Wright 1940). Each social group is an age‐structured unit where individuals mate according to different strategies (e.g., monogamy and polygyny) rather than at random. The model explicitly simulates diploid individuals, which are represented by a number of microsatellite markers evolving under the strict stepwise mutation model (SMM). At the beginning of the simulation, we create individuals by sampling genomes from a Wright–Fisher population with *θ* = 20 (where *θ* is the scaled mutation parameter *4 N* *μ* and *μ* is the per generation mutation rate for the entire locus). This Wright–Fisher population was generated using the ms program (Hudson 2002). In other words, we assume that the simulated population is funded by a few individuals from a large random mating population, a simplifying assumption that ensured that mutation–drift equilibrium was reached much quicker than if we had to wait for mutations to appear. We assumed a mutation rate equal to *μ = 5*e−4, which is of the order of magnitude measured for microsatellites (Sun et al. 2012; Whittaker et al. 2003).

Under this model, individuals undergo a simplified life cycle which encompasses four key stages: offspring, juveniles, and adults (reproductive and non‐reproductive, see below). Transitions between these stages occur depending on the age of an individual. Specifically, individuals are assumed to be offspring until they are weaned, juveniles if they are above weaning age but still cannot reproduce, and finally adults. Transition ages and other life‐history parameters are preset to given values according to the life cycle of a particular species of interest (see Table 1 for a detailed list of parameters). Note that under this framework, a social group is a relatively small age‐structured aggregation of kin‐related individuals. This is thus a model with overlapping generations.

**TABLE 1:** Life history parameters and values as used under the social group program simulations.

Parameter	Value
Life span	28 ticks (7 years)
Max. life span	40 ticks (10 years)
Infant mortality	0.3
#Offspring	2
Weaning age	1 tick (3 months)
Reproductive age	8 ticks (2 years)
Birth interval	4 ticks (1 year)

A time unit in the model (a tick) consists of elementary updates of the state of each individual (e.g., age and reproductive class). A tick corresponds to an arbitrary time unit which can be a year, a month, a day, or a few hours, depending on the life cycle of the species of interest. It is defined as the smallest time unit identifiable for the species as modeled in our simplified life cycle. At each tick, individuals age, die, reproduce, migrate, and colonize new groups (see below).

*Reproduction*: Although all adults above reproductive age can potentially mate, only a limited number of adults actually mates. These individuals are identified as reproductive status individuals (RS). This means that, although there are many adults (RS and non‐RS) within a group, mating pairs are formed within social groups among RS males and RS females only. The mating system is defined by the sex ratio of these RS individuals. For instance, under monogamy there is only one mating pair per social group (1♂:1♀), and under polygyny one single RS male mates with several females (1♂:10♀ in our simulations). In other words, as in a real species, few individuals control reproduction for consecutive mating seasons. This is for instance the case of many primate societies characterized by dominance hierarchies where dominant males enjoy exclusive access to females during an extensive period of time and until takeover by other males. The model assumes that an individual may reproduce until death, that is, once an individual becomes RS it may stay reproductive during its entire life. In practice, this means that the lifetime in the model can be seen as the reproductive lifetime of a real species.

At each reproduction event pairs are formed by randomly assigning one, and only one, RS male to each RS female. This implies that females reproduce with one single male although RS males can sire offspring from several RS females. The total number of offspring per social group is taken from a truncated Poisson distribution, constraining on the mean number of offspring + 1 per mating pair. This constraint derives from the fact that in mammals it is rare for a female to have an extremely large number of offspring.

*Dispersal*: Under the social groups simulation framework there is a difference between migration and colonization events. *Colonization* occurs when a new social group is established to replace a group that vanished (because all individuals died). In this situation, new RS individuals are chosen at random among all non‐RS individuals above reproductive age to establish a new social group. *Migration* occurs when individuals move to become RS in an already established group. It is a consequence of the death of some, but not all, RS individuals within a group, which creates “reproductive vacancies”. These vacancies are filled by migrants that are randomly chosen among non‐RS individuals living in other social groups. Alternatively, if one of the sexes is philopatric, RS individuals of that sex are replaced by non‐RS individuals randomly chosen from within their own social group. Note that migration is not defined by an explicit rate as in classical population genetics models, rather it is a consequence of the death rate as determined by the life span parameter.

The model includes two death parameters: infant and adult mortality. Infant mortality is a fixed value that indicates survival during the offspring stage, adult mortality is indirectly modeled by the lifespan parameter, which follows a truncated Poisson distribution. There is no carrying capacity or density regulation of any form in the model. Population size and viability (i.e., no population crash) are an indirect consequence of the relationship between infant and adult mortality, and birth rates. While some parameter combinations resulted in unrealistically large populations, others led to population crashes. We ran a few preliminary analyses and chose a parameter combination (Table 1) resulting in both viable populations and population sizes within acceptable values for real populations. For instance, under the final combination of parameters, populations of 50 social groups present hundreds to thousands of individuals, with low to moderate genetic diversity. Specifically, the total census population size under monogamy was around 600 individuals. For the other mating systems, it led to larger numbers of individuals, around 1000 individuals under polygynandry and 6000 under polygyny.

### 2.2 Simulations Under the Social Groups Model

For this study, we considered three mating systems: monogamy, polygyny, and polygynandry defined by the ratio RS♂:RS♀: 1:1 in monogamy, 1:10 in polygyny, and 2:2 in polygynandry (Table 2). We assumed that females are philopatric, that is only males disperse within the network of social groups. This is because mammalian social systems are typically characterized by female philopatry and male dispersal (Greenwood 1980; Johnson and Gaines 1990). Life‐history parameters were assumed as in Table 1. Simulations were run for 5e5 ticks, corresponding to > 25,000 generations, much beyond that required to attain mutation–drift equilibrium. Genetic and demographic equilibrium were confirmed based on the convergence of summary statistics, specifically the expected heterozygosity, *H*_e_ and the *F*_ST_ (Nei 1977; Wright 1951). For each scenario, we produced 100 independent replicates.

**TABLE 2:** Scenarios simulated in this study.

Framework	Mating	Sex ratio	#SG	Sex system	Ploidy	*θ*
Social groups	Monogamy	1:1	10,50,500	Dioecious	2*n*	—
Social groups	Polygynandry	2:2	10,50,500	Dioecious	2*n*	—
Social groups	Polygyny	1:10	10,50,500	Dioecious	2*n*	—
EASYPOP	Random	1:1	1000	Dioecious	2*n*	—
EASYPOP	Random	1:1	2000	—	*n*	—
ms	Random	1:1	—	—	*n*	(0.1–1), 1.5, 2

Abbreviations: #SG, number of social groups; *θ*, the scaled mutation parameter *4Nμ*, where *μ* is the per generation mutation rate for the entire locus.

We simulated datasets under 10, 50, and 500 social groups. Although only RS individuals reproduce, populations comprise many other individuals, non‐reproductive adults, juveniles, and offspring that are part of the census size. While populations of 10 social groups correspond to census sizes of a few hundred to thousands of individuals with very little diversity, 50 social groups hold hundreds to thousands of individuals (census size) and low to moderate genetic diversity. These values of diversity are within the range found for many species (Ghazi et al. 2021; Modi et al. 2021; Mukesh et al. 2015; Srinivas and Jhala 2024). On the other hand, 500 groups correspond to large census sizes of thousands to hundreds of thousands of individuals and high diversity. Note that simulated populations are stationary—certainly suffering neither collapse nor expansion—and their dynamics and diversity are maintained for thousands of generations.

For the demographic inference analysis, we sampled individuals alive at the last time point of the simulation. These were sampled at random among offspring, juveniles, RS individuals and non‐RS females to represent sampling individuals living in groups (thus excluding individuals that are not part of a group). Non‐RS males were not sampled as these correspond to individuals that would have dispersed in a real species and are thus not considered as part of a group.

Because the sampling strategy is known to have an impact on the detection of demographic signals, we used three different sampling strategies. We randomly sampled 20 individuals (i) from a small pool of five groups; (ii) from a larger pool of 10 (out of 10), 30 (out of 50), and 100 (out of 500) groups, as to mimic sampling being limited to part of the overall distribution of a population, and (iii) making sure each individual comes from a different social group, that is, one individual per group, as to avoid sampling kin related individuals. Note that when restricting to one individual per group, we could only sample 10 individuals under the 10 social groups scenario.

### 2.3 Simulations Under Panmictic Populations

We generated data under a single panmitic (random‐mating) non‐structured, constant‐size population. This allowed us to obtain expectations for idealized populations. We simulated coalescent genealogies under the ms program (Hudson 2002) and translated them into microsatellite length variation data assuming the SMM, using the microsat program (Hudson 2002). We generated data under a wide range of *θ* values. *θ* varied between 0.1 and 1 in increments of 0.1 and also took values of 1.5 and 2. This generated datasets within the range of *H*_e_ values obtained under the social groups simulations. We also generated data under EASYPOP, an individual‐based forward time program to simulate microsatellite genetic data under the SMM mutation model (Balloux 2001). We simulated one single population of 1000 diploid dioecious individuals (500♂ and 500♀) and also one population of 2000 haploid individuals. As in the social groups' simulations, we simulated 100 loci under a mutation rate of *μ =* 5e‐4 per locus per generation. We generated 100 independent datasets under each scenario. We sampled 20 diploid individuals in the EASYPOP and 40 genes under ms at the last time point of each simulation to carry out the demographic analyses. Note that the EASYPOP and the social group programs are forward in time and explicitly represent all individuals living in a population, whereas the ms is a coalescent‐based program.

In total, we generated data under 23 different scenarios: social groups monogamy, polygyny, and polygynandry with (10; 50; 500) groups (a total of nine scenarios), ms *θ* = (0.1‐1), 1.5, 2 (in a total of 12 scenarios) and EASYPOP haploid and diploid (two scenarios; see Table 2).

### 2.4 Inference of the Demographic History

The simulated datasets were analyzed using two methods to detect population size changes: BOTTLENECK v.1.2.1 (Piry, Luikart, and Cornuet 1999) and msvar 0.4 (Beaumont 1999).

*BOTTLENECK*: The BOTTLENECK software (Piry, Luikart, and Cornuet 1999) implements a simple method that allows detect population size changes, either decreases or expansions, based on a heterozygosity excess test (Cornuet and Luikart 1996). It tests for the null hypothesis of a stationary population by identifying deviations from the mutation–drift equilibrium. In a population with constant size, the forces of mutation and drift balance each other maintaining genetic diversity at an equilibrium: the mutation–drift equilibrium. Ewens (1972) has shown that under this equilibrium the number of alleles in a sample is sufficient to calculate the theoretical expected distribution of the heterozygosity. The heterozygosity test as implemented in BOTTLENECK computes this theoretical heterozygosity by simulating coalescent trees under a given mutation model (the SMM in this study), and compares it to the expected heterozygosity (*H*_e_ Nei's gene diversity; Nei 1978). While an excess of heterozygosity provides evidence for population contraction, a deficit provides evidence for expansion. We conducted 1000 coalescent simulations assuming the SMM. Note that the same mutation model was used in the social groups, EASYPOP, and ms programs. Statistical significance was tested using the Wilcoxon signed‐rank test (Cornuet and Luikart 1996). We considered an arbitrary *p*‐value < 5% as evidence of support for a population size change. Tests were performed using 100 independent loci and 100 independent datasets for each scenario.

Under the BOTTLENECK program, we analyzed data from 50 social groups and ms data with *θ =* 2, corresponding to moderate to high values of diversity.

*msvar*: The msvar software implements a likelihood‐based Bayesian method (Beaumont 1999). This method analyses microsatellite data evolving according to the SMM. It assumes that at *T*_a_ generations ago one single stable population changed from an ancestral size *N*_1_ to a current size *N*_0_, either exponentially or linearly. The method estimates the posterior distribution of the model parameters—the ratio *N*_0_/*N*_1_, the scaled time *T*_f_ = *T*_a_/*N*_0_, and the scaled mutation rate *μ* under a simple model of size change. It uses the full allelic distribution of a population, specifically allelic states, and the allelic frequency distribution. We assumed an exponential change and we set priors as wide uniform distributions varying between (−5, 5) for the three parameters *log*_10_ (*r*), *log*_10_ (*μ*), and *log*_10_ (*T*_a_). Because when populations are subdivided into demes (random‐mating units) a false signal of population bottleneck can be observed (Wakeley 1999), we chose positive *log*_10_ (*r*) values as starting points for the MCMC, corresponding to expansions, far from the expected bottleneck. However, because we ended up with positive posteriors particularly strong under some scenarios, results were confirmed by reanalyzing some runs using negative *log*_10_ (*r*) as starting values for the MCMC. The initial *μ* values were chosen for each locus from a uniform (0,1) distribution.

For computational reasons, we analyzed a subset of 20 datasets in msvar. For each scenario, we analyzed 20 independent datasets and 20 loci. We performed a single long run of 5 × 100 steps with a thinning interval of 50,000 steps. The convergence of the chain was checked visually and the first 10% of the chain was discarded as burn‐in. The number of steps performed was enough to reach the stationary distribution and the few datasets in which chains did not seem to converge were discarded.

### 2.5 Estimation of Contemporary *N*_e_

We estimated *N*_e_ of the simulated populations using the NeEstimator (version 2.1.; Do et al. 2014). This software estimates contemporary *N*_e_ using three methods: patterns of linkage disequilibrium (LD) between unlinked markers, heterozygosity excess, and molecular coancestry. We estimated *N*_e_ under the LD method (Waples 2006). This method uses Weir's unbiased estimator of *Δ* which calculates the correlation of allele frequencies at each pair of loci (*Δ* ^2^), where the expected *N*_e_ is a function of *Δ* ^2^, sample size, recombination rate, and mating system, using statistics corrected by Do et al. (2014) and derived by Waples (2006) for random mating and monogamy.

We used two sampling strategies whereby we sampled 20, 50, and 100 individuals either: (i) at random among the 10 (out of 10), 30 (out of 50), and 100 (out of 500) social groups or (ii) by making sure that each sampled individual came from a different group. This is intended to minimize the relatedness within samples. Again, note that when sampling one individual per group the sample size was necessarily limited to the total number of groups—10 individuals when 10 groups were simulated and 50 individuals for simulations with 50 groups. For each independent simulation, we obtained 10 independent sample replicates. We estimated *N*_e_ using 20 microsatellite loci, a number comparable to what is used in real datasets (Goossens et al. 2006; Lester et al. 2021; Quéméré et al. 2012). The NeEstimator program specifies one of two options, “random mating” or “monogamy” and therefore we choose monogamous mating whenever samples were obtained under the monogamous scenario. Once the presence of rare alleles affects the performance of the LD method, we excluded alleles that occurred as one single copy in the sample.

## 3 Results

### 3.1 Demographic Inference With BOTTLENECK

**FIGURE 1:**
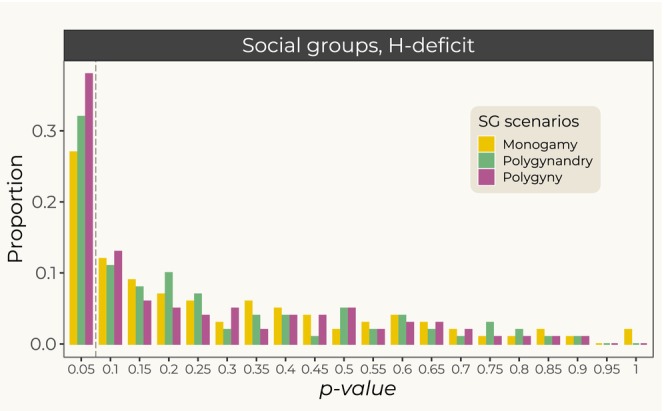
Detection of change in population size in social groups in BOTTLENECK. Distribution of *p*‐values obtained under the *H*‐deficit test providing evidence for expansions. The dashed vertical line indicates *p*‐value = 0.05, under which only 5% of datasets are expected to be found by chance.

The BOTTLENECK method detected departures from mutation–drift equilibrium in many datasets simulated under the social structure model. A significant deficit of heterozygosity (*H*‐deficit, *p*‐value < 0.05), corresponding to expansions, was detected in 27% datasets simulated under monogamy, 32% under polygynandry, and 38% under polygyny (Figure 1). As these proportions show, we observed some differences between mating systems but the general signal was a consistent difference from what is expected under classical population genetics models. Classical population structure is expected to exhibit bottleneck signals, that is, a significant heterozygosity excess (*H*‐excess). In our simulations, the support for bottlenecks was poor and only two of the 300 datasets analyzed showed significant *H*‐excess (Figure S1). These datasets corresponded to monogamy scenarios but it is unclear if this should be taken at face value. Altogether, the *p*‐value distributions were asymmetrical with higher probabilities for quantiles corresponding to *H*‐deficits (Figure 1). In other words, all scenarios showed similar biases toward spurious signals of expansion.

We also detected false signals of population size changes in datasets simulated under EASYPOP (Wright–Fisher panmictic models, Figure S2). BOTTLENECK detected spurious expansions (a significant *H*‐deficit) in 37% of datasets in the haploid model and in 29% of datasets in the diploid model. Note that in the haploid scenario, 66% of all datasets were highly biased in either direction (37% significant for expansion and 29% for a bottleneck). A bias toward spurious expansions was also found in datasets simulated under the ms coalescent‐based simulation program (*θ* = 2), *p‐*values were significant for expansions in 30% of the datasets (*H‐*deficit). Thus, with the exception of the EASYPOP haploid scenario, for which many datasets favored bottlenecks (Figure S2 lower panel), BOTTLENECK was biased toward expansions even under panmictic models. Note that under panmictic models, BOTTLENECK should either detect no signal of departure from equilibrium or similar proportions of false positives in both directions (bottlenecks and expansions).

### 3.2 Demographic Inference in msvar

**FIGURE 2:**
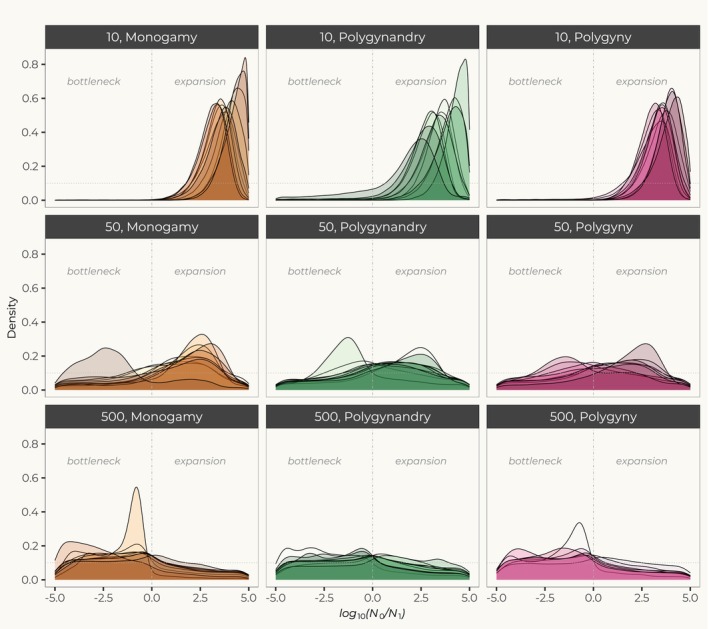
Detection of change in population size in msvar. Posterior distributions for log_10_ (*r*), the ratio of present *N*_0_ over past *N*_1_ population size. Results were obtained from sampling individuals at random from a large pool of social groups. 10, 30, and 100 groups were sampled from a total of 10, 50, and 500 groups. The prior for *log*_10_ (*r*), set as a uniform between −5 and 5, is represented by the horizontal dotted line.

In datasets generated under the social groups framework, msvar often inferred spurious population size changes. These were frequently toward expansions, although spurious signals of contractions were also found. For simulations with 10 and 50 social groups, the sampling strategy did not seem to influence estimated changes in *N*_e_. In all scenarios, except monogamy (see below), general conclusions about demographic history were comparable among sampling schemes (Figure 2, and Figures S3, S4). Under simulations with 10 groups, we consistently found strong spurious expansion signals: the *log*_10_ (*r*) posteriors were biased toward positive values across all social systems (Figure 2, and Figures S3, S4 top panels). Among scenarios with 50 social groups, signals of population size change varied widely across simulations. Overall, the marginal posterior distributions of *log*_10_ (*r*) *= N*_0_ */N*_1_ were wide and relatively flat, suggesting a stationary model without very strong signals for increasing or decreasing populations (Figure 2 and Figure S3 middle panels). However, in 50 groups scenarios, when a spurious demographic signal was detected this was often biased toward an expansion. Particularly, monogamy led to unexpectedly strong spurious expansion signals that were magnified when only one individual per group was sampled (Figure S4).

Under 500 groups scenarios, the sampling strategy had an effect in the detection of population size changes (Figures 2, and Figures S3, S4). First, in 500 groups, we did not find signals of population size change when we sampled from a large pool of social groups either at random or from one individual per group. Most posteriors were flat and did not differ from priors (Figure 2 and Figure S4). However, posteriors were consistently biased toward bottlenecks when we sampled individuals from a smaller pool of five social groups (Figure S3 lower panels). These disparate results are in agreement with expectations for deme‐structured models—when samples derive from a single deme, bottlenecks are expected. However, the effect of the structure is minimized when each individual comes from a different deme (Chikhi et al. 2010). Still, when we found a bottleneck, this did not seem as strong as those found in deme‐based models.

Overall, it appears that at intermediate levels of diversity demographic signals are the result of an interplay between genetic diversity, the social structure, and the sampling scheme. These intermediate levels of diversity (Figure S5) are within those observed in real datasets from endangered species (Ghazi et al. 2021; Modi et al. 2021; Mukesh et al. 2015; Srinivas and Jhala 2024). At the same time, msvar detected spurious expansions as a consequence of the limited level of genetic diversity caused by a very small number of social groups, whereas when we increased the number of groups, and diversity, the signal moved toward classical expectations for structured populations (Figure 2).

Diversity seems to play a major role in creating a spurious bias toward expansions. Spurious expansions were also detected in datasets simulated under the ms scenarios with low to moderate diversity (Figures S6 and S7). msvar inferred posteriors highly biased toward positive values under ms *θ* = (0.1–0.5). However, at higher levels of diversity (*θ* >1) msvar did not detect population size changes, as expected for Wright–Fisher scenarios (Figure S6).

Surprisingly, a panmictic diploid forward model (EASYPOP) led to spurious bottlenecks (Figure S8). For datasets simulated under a panmitic haploid unstructured population (EASYPOP), posteriors of *log*_10_ (*r*) were flat and very similar to the priors (Figure S8). Our results suggest that the detection of population size changes is a complex function of diversity and population structure, including social structure (mating and dispersal) and sampling.

### 3.3 Contemporary *N*_e_ Estimates

**FIGURE 3:**
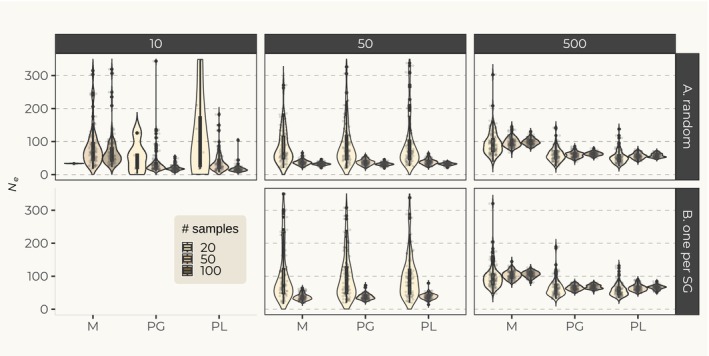
Effective size (*N*_e_) estimates. Each panel corresponds to one mating system as simulated under the social groups model. Each color corresponds to a number of sampled individuals, *n* = 20, 50, and 100. In (A) individuals were sampled at random and in (B) one single individual was sampled per group. Point estimates that returned infinite values were removed. For that reason, all estimates for 10 social groups sampling scheme B and most values for 10 groups sampling scheme A, 20 samples (lighter color) are omitted. The *y*‐axis is truncated at 350. M, Monogamy; PG, Polygynandry; PL, Polygyny.

Figure 3 shows the distribution of *N*_e_ estimates for the three mating systems, under different sampling schemes and sample sizes. As expected we found that the variance in *N*_e_ estimates decreased with the number of sampled individuals for all sampling schemes and mating systems. In particular, the variance in *N*_e_ estimates was particularly high when we sampled 20 individuals, including a few values as high as 2000 and infinite estimates. These infinite values are expected under small sample sizes and are discussed by Waples and Do (2008). The corresponding datasets were removed and omitted from the plots. This is why we do not show the results for 10 social groups when only one single individual is sampled per group. While small sample sizes could return confusing *N*_e_ estimates, we found that sample sizes above 50 returned consistent mean *N*_e_ estimates. With 50 and 500 social groups, we found that the two sampling schemes (one individual per group or sampling at random), led to similar *N*_e_ values (Figure 3). Thus, these results suggest that for large sample sizes, NeEstimator provided consistent estimates for a particular scenario and sampling scheme with little variation across sampling schemes. Interestingly, the *N*_e_ estimates were similar for the simulation with 50 groups for the different mating systems, whereas for 500 groups the estimates were higher under monogamy.

We also found that the estimated *N*_e_ was much lower than the number of RS individuals. This was observed under the three simulated mating systems (Figure S9). While estimates with 50 social groups returned *N*_e_ values around 30, the actual number of RS was above 100 in all simulations. With 500 social groups mean *N*_e_ estimates were below 150, while the number of RS was above 1000 in all mating systems (Figure S9). We stress here that the total number of individuals simulated is much larger than the number of RS individuals actively reproducing. It also includes adults who never become RS in a social group, juveniles, and infants. Also, note that the number of RS individuals is not, and should not be, interpreted as a proxy for *N*_e_.

## 4 Discussion

Our results suggest that when populations are socially structured, some methods that test for changes in population size or estimate current *N*_e_ can produce results that are difficult to interpret in relation to the actual demography of the population. As a consequence, these methods are potentially misleading when identifying trajectories in terms of changes in *N*_e_. We focused on three approaches widely used in conservation genetics, and that directly or indirectly infer and interpret *N*_e_. The first approach, BOTTLENECK, identifies departures from mutation–drift equilibrium that are usually interpreted in terms of bottlenecks or expansion. The second approach, msvar dates and quantifies population size changes by estimating the ratio of current to past *N*_e_. BOTTLENECK can be seen as a way to test whether one single *N*_e_ value can be used to model the population of interest, while msvar goes one step further by fitting a two‐*N*_e_ model. The third method, NeEstimator, directly estimates one contemporary *N*_e_ based on patterns of LD across independent genetic markers.

Our objective was to improve our understanding of the concept of *N*_e_ when populations or species are organized in social groups under various mating systems. We identified many cases where no single *N*_e_ could meaningfully explain the data (BOTTLENECK results) and where contradictory two *N*_e_ models would be inferred (msvar results). We also found that the NeEstimator inferred *N*_e_ values very far from the simulated population sizes, whether we consider the number of RS individuals or the census size, *N*_c_.

The methods we used here have been widely applied to endangered species. Low current effective sizes have been often estimated and changes in *N*_e_ often detected (Goossens et al. 2006; Quéméré et al. 2012; Storz and Beaumont 2002). For instance, bottlenecks were detected in orangutans from Sabah and lemurs in Madagascar when using the msvar and BOTTLENECK methods (Goossens et al. 2006; Quéméré et al. 2012). In these studies, the authors have tested for the dependence of apparent bottlenecks on population structure, but they did not test for the effect of social structure. Jana and Karanth (2023) used BOTTLENECK and found a recent expansion in blackbucks (the Indian antelope *Antilope cervicapra*), suggesting evidence of species adaptation to human‐altered landscapes. Although the Indian blackbuck may be indeed expanding, we emphasize that the expansion signal could in part be the result of social structure or change in social structure, rather than the result of a reduction in conservation threats. Further, mating systems and social structure have an effect on the *N*_c_ */N*_e_ ratio. Under most scenarios of social structure, high ratios of *N*_c_ */N*_e_ can be observed. A number of recent studies have stressed that when *N_c_* >> *N*_e_, some measures of genetic diversity, such as allelic richness, may be higher than that predicted by the *N*_e_ estimates as obtained using statistics such as *H*_e_ (Allendorf, Hössjer, and Ryman 2024; Mergeay 2024). This too could lead to an apparent departure from equilibrium for methods such as those implemented in BOTTLENECK as they implicitly compare *N*_e_ estimates based on the number of alleles and *H*_e_. More work should be done from the theoretical point of view to determine whether and how the *N*_c_ */N*_e_ ratio must be accounted for in conservation.

Our simulation study suggests that, under the three mating systems tested (monogamy, polygynandry, and polygyny), social structure can lead to the detection of spurious *N*_e_ change estimates. We found that the direction of the demographic signal (decline or expansion) changed as a function of the number of social groups simulated, the mating system, and the sampling scheme. In particular, populations composed of fewer groups showed a spurious but strong expansion, whereas the signal was lost/shifted toward contraction when the number of social groups increased. The “expansion bias” might mislead conservationists about species for which conservation actions are mostly important. The “bottleneck bias” converges toward what has been observed in structured populations where population structure is modeled by panmictic demes connected by gene flow (i.e., *n*‐island or stepping‐stone models).

Population structure has been studied extensively in the last decades (Maruyama and Kimura 1980; Pannell and Charlesworth 2000; Slatkin 1977; Wakeley 1999; Whitlock 1992; Wright 1931). A number of theoretical studies have proposed different analytical equations to compute *N*_e_ under structured models (see Charlesworth 2009; Charlesworth, Charlesworth, and Barton 2003; Sjödin et al. 2005; Wakeley 1999). In addition, studies have suggested simplifications to deal with structure, such as sex differences and age structure under the assumption that reproduction and migration do not have long‐lasting effects and can be averaged out using fast time‐scale approximations. These assumptions implicitly mean that social structure is unlikely to be very important in explaining patterns of genetic diversity. That is, it does not represent a problem to compute one single *N*_e_ for socially structured populations. Similarly, methods for inferring changes in *N*_e_ assume that the effects of structure are negligible compared to changes in population size (Beaumont 1999; Li and Durbin 2011; Liu and Fu 2020). However, more than two decades of research have shown that when samples are drawn from structured populations, these methods will detect, date, and quantify changes in *N*_e_ that never occurred (Chikhi et al. 2010; Heller, Chikhi, and Siegismund 2013; Hoban et al. 2013; Mazet et al. 2016; Paz‐Vinas et al. 2013; Städler et al. 2009; Wakeley 1999). Inferences are often biased toward the detection of recent bottlenecks in coalescent‐based methods, with stronger effects when samples come from a single deme exchanging migrants with other demes (Chikhi et al. 2010; Mazet et al. 2016; Wakeley 1999). Moreover, a method inferring changes in *N*_e_ will infer a current *N*_e_ close to the deme size and an ancient *N*_e_ close to the overall (large) population size. Wakeley (1999) noted that when individuals are taken from several demes this bottleneck signal should disappear as the gene tree of this “global” sample will tend toward that of a single *N*_e_ of the metapopulation size. But this result may not hold when social structure is present.

The expectations for the classical structure, above, are valid under the assumption of constant size (no change in migration, deme size, or number of demes) and symmetry (migration and deme size, as in the *n*‐island). If there are changes in deme size and migration patterns, more complex histories of *N*_e_ can be inferred. For instance, expansions can actually be found in populations experiencing asymmetrical gene flow (Paz‐Vinas et al. 2013), or when sampling two haploid genomes from different demes (Mazet et al. 2016; see also Chikhi et al. 2018 for different asymmetrical models such as continent‐island models). Still, nearly no work has tried to understand the effect of social structure. One natural simplification might be to consider that social units are like small demes and apply the structured coalescent of Notohara (1990) and Wilkinson‐Herbots (1998). However, our results suggest that this may not be enough when populations are socially structured. While the ancestral coalescence process should provide a good approximation under a variety of departures from the Wright–Fisher models, such as two‐sexes, age structure, and mating systems (Möhle 1998, 2000; Wakeley 2009), this principle relies on strong assumptions of (i) a very large number of islands (D → ∞), (ii) large deme size (N → ∞), (iii) “strong migration,” and (iv) small sample size compared to deme size (s < < N). Social groups will rarely follow any of these approximations. In social species, sample sizes are usually of the same order of magnitude as the group size. Therefore, in a single time unit, multiple coalescent events may occur. Under continuous time coalescence, multiple coalescences can never take place. Also, in real populations genealogies are constrained by pedigrees. For that reason, gene genealogies can deviate from expected standard coalescent in the very recent past (e.g., the last 10 generations, Wakeley et al. 2012), when many coalescences are expected to occur quickly. But, under some modes of mating, the actual pedigree prevents coalescent events in the very few past generations. For example, when females are philopatric and males migrate, parents and offspring can never share a common ancestor in the male lineage in the immediate previous generation. Moreover, in social groups individuals have varying degrees of relatedness (sibs, half‐sibs, parent‐offspring, etc.). Sampling from such diversity can result in different genealogies and variable *N*_e_ estimates. This could explain why both bottlenecks and expansions can be inferred under the same mating scenario.

It thus appears that social groups, as simulated here, have properties that violate the assumptions required to observe the expected convergence toward the standard coalescent model. We must stress though that such departures do not seem to be limited to social structure. For instance, simulations with ms and EASYPOP showed that Wright–Fisher models could generate surprising expansion or bottleneck signals when *θ*, the scaled mutation rate, was low. Cornuet and Luikart (1996) noted, some 30 years ago, a series of conditions that may lead to spurious expansion signals when testing for deviations from mutation–drift equilibrium. These are hidden substructure, recent immigration, and sampling hybrids of two populations. All these cases may occur when samples come from populations subdivided into social groups and thus, it is not surprising that BOTTLENECK detects expansions. While these situations may seem purely theoretical, the recent study by Jana and Karanth (2023), detecting an expansion in endangered blackbucks, shows that it can actually have practical conservation implications. Here we focused on microsatellite markers, which remain prevalent for species where non‐invasive sampling is necessary. However, the effect of social structure likely extends to all coalescent‐based inference methods, including those based on whole‐genome data such as site‐frequency‐spectrum or haplotype data (Fournier et al. 2023; Gutenkunst et al. 2009). These data provide significantly more information than microsatellite data and may thus be very useful for reconstructing the recent evolutionary history of species (Li and Durbin 2011; Novo et al. 2023), but they may also provide misleading support for events that never took place (Chikhi et al. 2010; Mazet et al. 2016). It would thus be important to study the potentially spurious effects caused by social structure also in genome‐wide data, that are now more frequently employed for endangered species (e.g. Guevara et al. 2021 on sifakas; Prado‐Martinez et al. 2013 on chimpanzees; Teixeira et al. 2021 on mouse lemurs).

### 4.1 Final Remarks

The concept of *N*_e_ was created to compare different biological models and provide a common scale for measuring genetic drift in natural populations. Depending on the properties of interest (temporal variance in allele frequencies or total amount of diversity) *N*_e_ estimates can have different values. With the advent of the coalescent theory, the concept of coalescent *N*_e_ was developed, but it soon appeared that it was problematic too when populations were structured (Sjödin et al. 2005). As we already noted, no constant‐size population (i.e., no single *N*_e_) can explain the distribution of gene genealogies under a structured model. Even in an *n*‐island model, a “dynamic *N*_e_” is required, as a constant *N*_e_ cannot adequately explain the data leading to notions related to a local versus global *N*_e_ (Mazet et al. 2016; Novo et al. 2023; Ryman, Laikre, and Hössjer 2019; Tenesa et al. 2007; Wakeley 1999). Here, we show that the structure created by mating systems may create different biases that may be negligible for some questions and central to others. It is one of the challenges that population and conservation biologists will have to face. In addition, as soon as a model is structured the genealogies will depend on the sampling scheme. The concept of *N*_e_ is supposed to characterize a stationary demographic model and should not depend on the sampling scheme. If depending on the sampling scheme we can infer two (or more) values of *N*_e_ it seems rather odd to claim that the concept of *N*_e_ is meaningful. Rather, one should use that result to try and improve our understanding of the population structure of the species we wish to protect. Their structure likely influenced its evolutionary history.

While we question the uncritical use of *N*_e_ for (socially and deme) structured populations, we stress that *N*_e_ is a useful concept. Computing and using *N*_e_ estimates remains necessary to compare models and inferences. It remains a central concept in population and conservation genetics. We must however acknowledge that there are many ways to compute *N*_e_ and not all are easily comparable. For conservation purposes, we should try to focus on methods that are more influenced by the recent history of species, and thus may better integrate the recent threats and be useful for conservation plans (see for instance Waples 2024 this issue). Although the coalescent *N*_e_ concept may be seen as less relevant for conservation, we think that all *N*_e_ concepts should be integrated to provide a more complete understanding of both the recent and more ancient history of endangered species. It is such a long‐term and short‐term view that should in the end be used for designing conservation actions.

## Conflicts of Interest

The authors declare no conflicts of interest.

## Supporting information


Data S1.

## Data Availability

The social groups simulations were performed using a framework developed by B.R.P. in Parreira and Chikhi (2015). The model was coded in the C++ language and is publicly available at: https://github.com/bparreir/Social‐Groups‐Simulations GitHub repository.
