# Supplementary material for: Effects of Social Structure on Effective Population Size Change Estimates
Source: Evol Appl. 2025 Jan 14;18(1):e70063. doi: 10.1111/eva.70063 (PMC11732743; doi:10.1111/eva.70063)
Supplement: Supplementary file 1 — Data S1. [file EVA-18-e70063-s001.pdf]

Supplementary Material for

Effects of social structure on effective population size change estimates

Bárbara Parreira, Shyam Gopalakrishnan, Lounès Chikhi

December 23, 2024

Corresponding author: barbara.parreira@sund.ku.dk;

1 Supplementary Figures

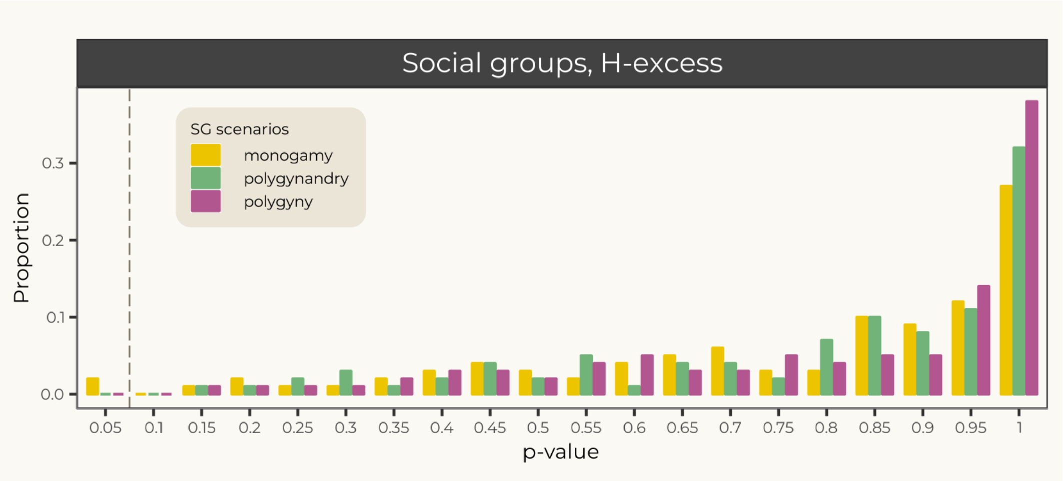

Figure S1: Detection of population size changes with the BOTTLENECK software. Data generated under the social groups software. Distribution of p-values obtained under the H-excess test. The dashed vertical line indicates p-value=0.05, under which only 5% of datasets are expected to be found by chance.

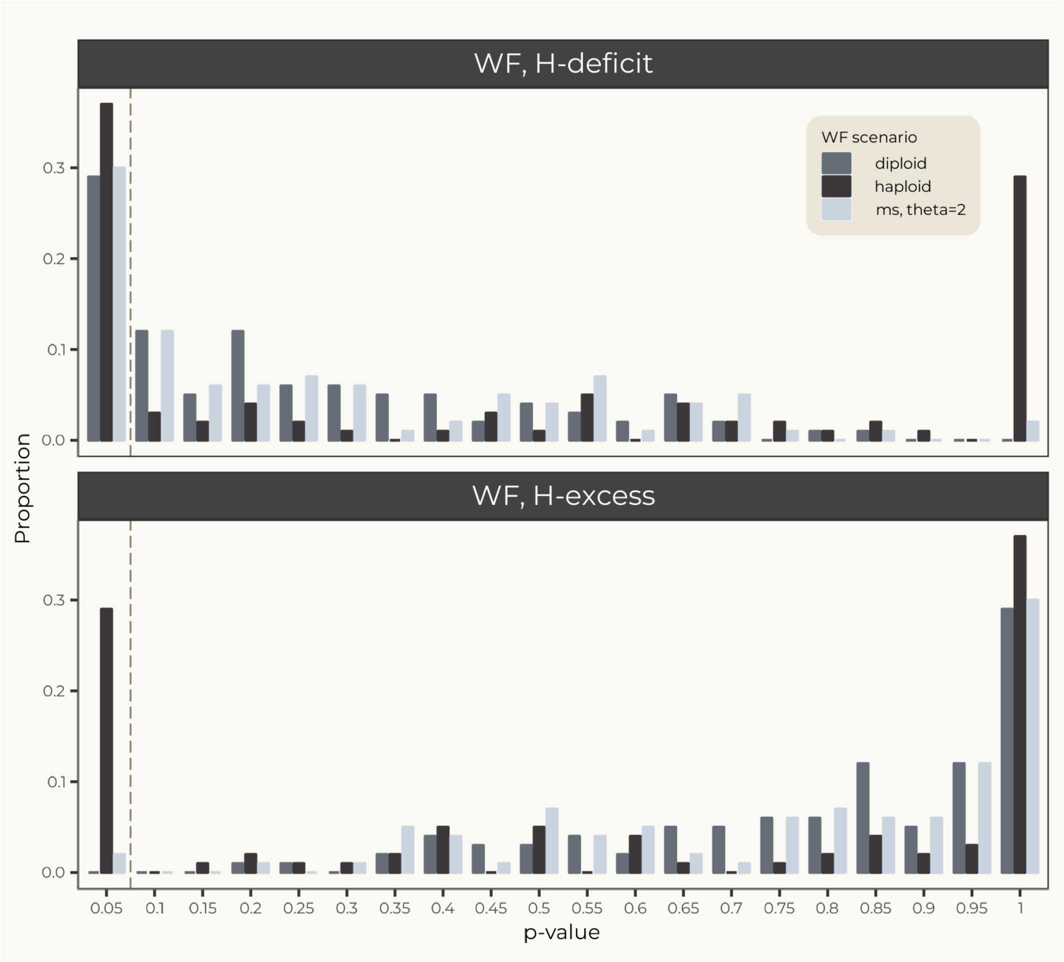

Figure S2: Detection of population size changes in panmitic populations scenarios under the BOTTLENECK software. Distribution of p-values obtained under the H-deficit and H-excess tests which provide evidence for expansions and bottlenecks, respectively. The dashed vertical line indicates p-value=0.05, under which only 5% of datasets are expected to be found by chance.

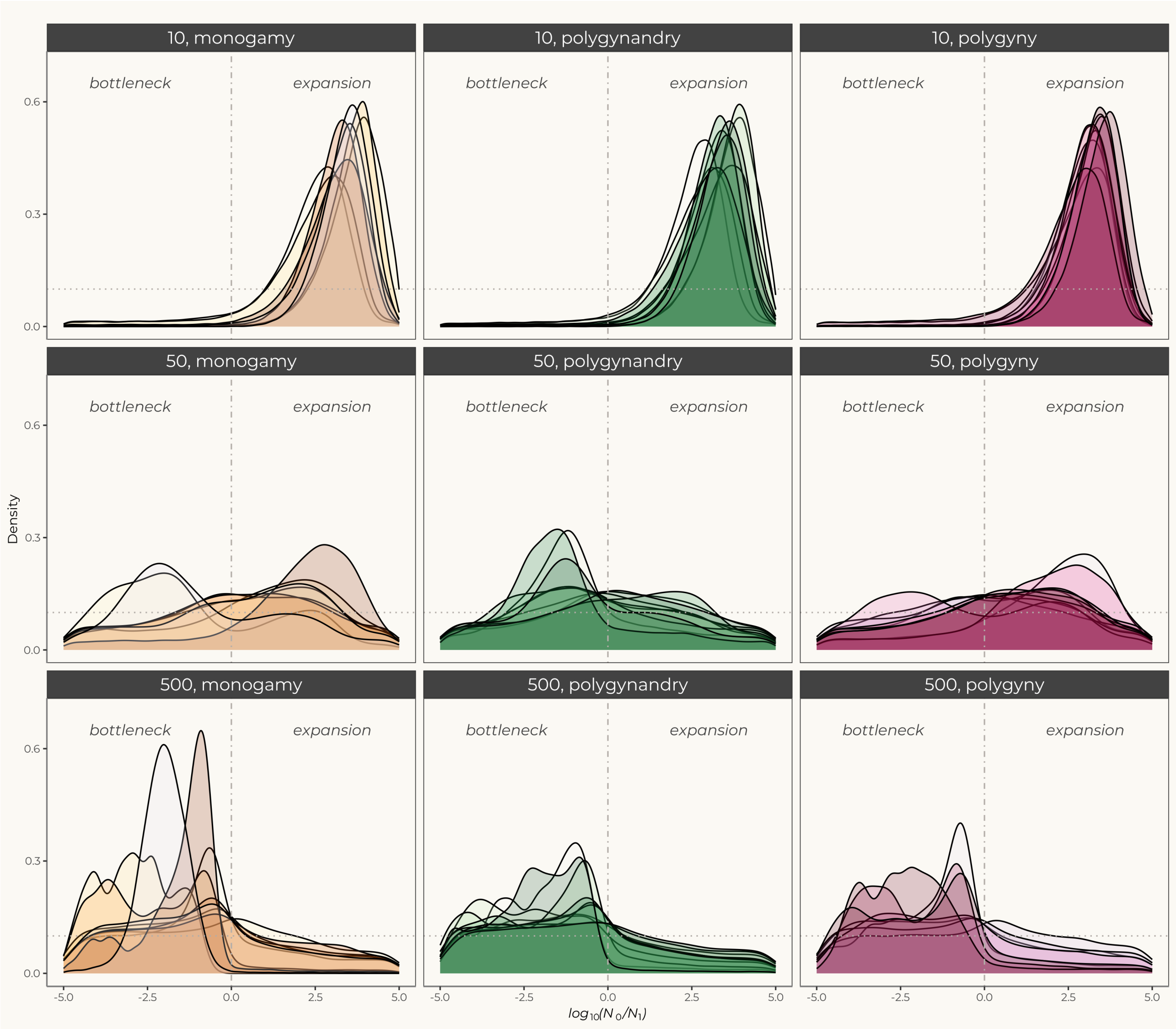

Figure S3: Detection of population size changes with msvar. Datasets obtained under the social groups program, individuals were sampled at random from a pool of five social groups. Posterior distributions for log_{10}(r), the ratio of present (N_0) over past (N_1) population size.

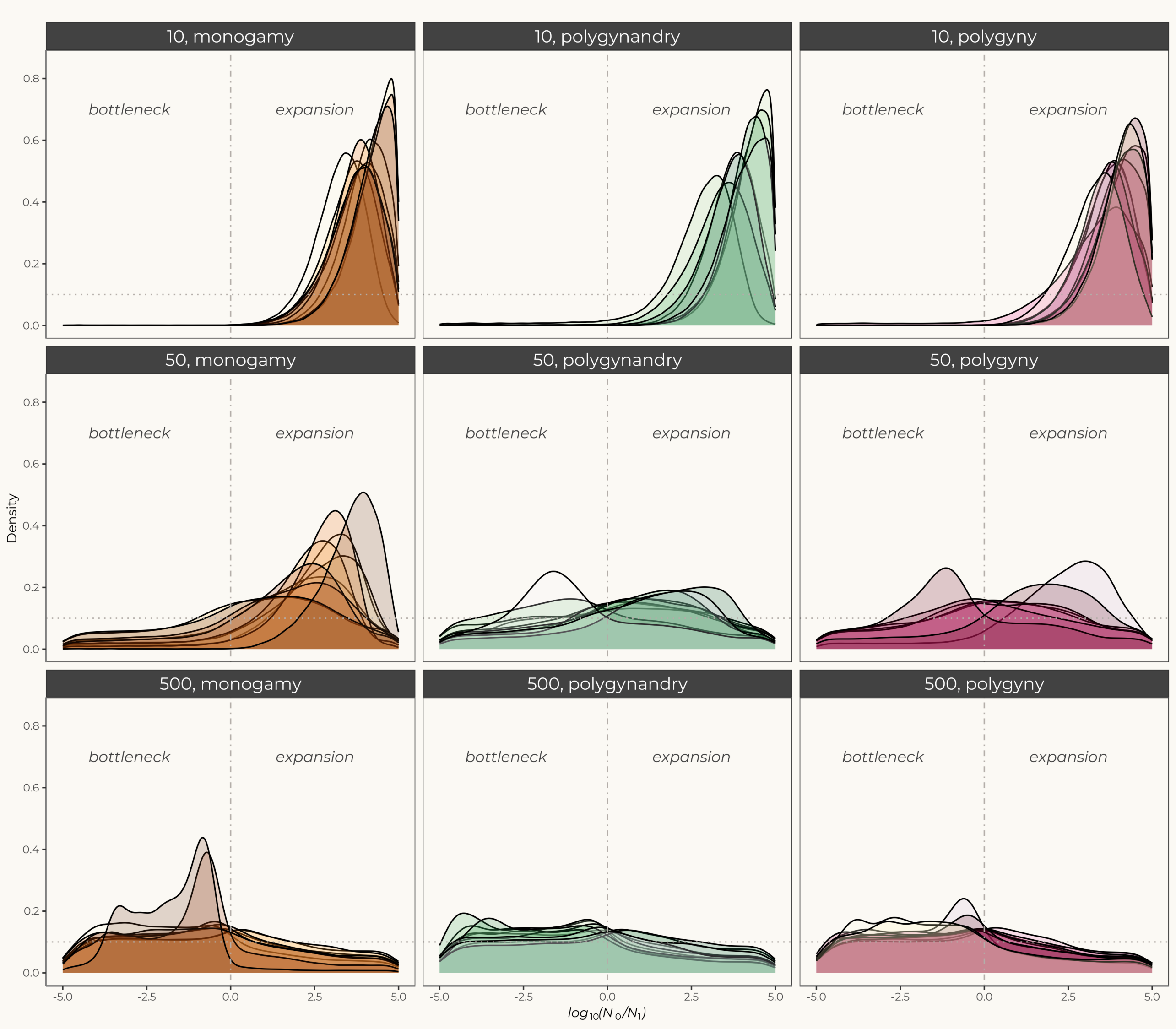

Figure S4: Detection of population size changes with msvar. Datasets obtained under the social groups programs, one individual was sampled per social group. Posterior distributions for $log_{10}(r)$, the ratio of present (N_0) over past (N_1) population size.

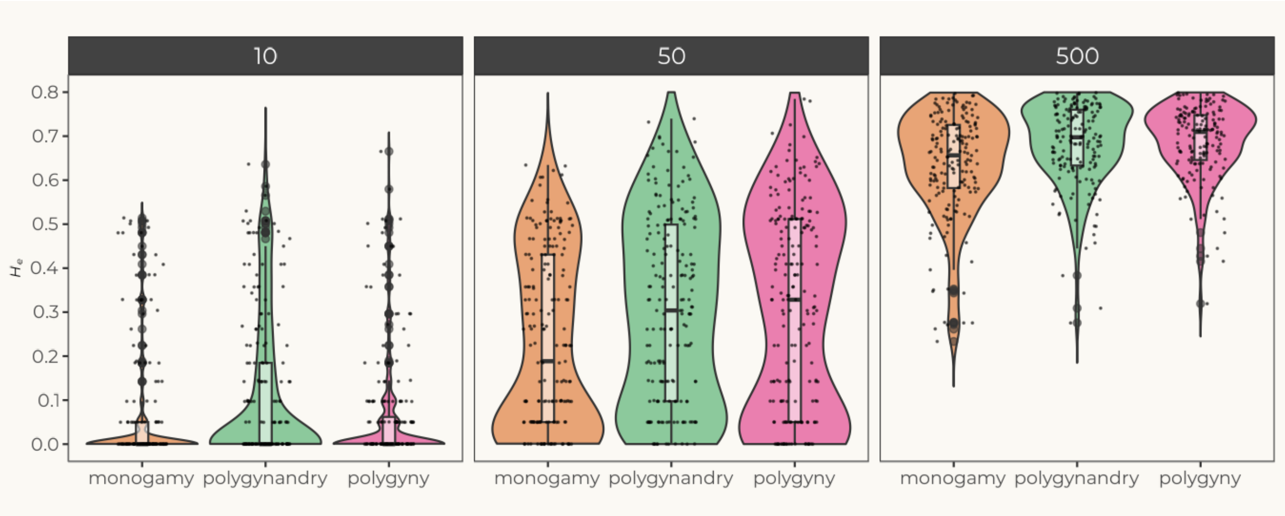

Figure S5: Distribution of expected heterozygosity, H_e obtained under the simulated social groups. Each dot is H_e estimated for one locus, according to Nei, 1978. Note that scenarios under ten and 50 groups hold low to moderate diversity and 500 groups lead to very high levels of diversity.

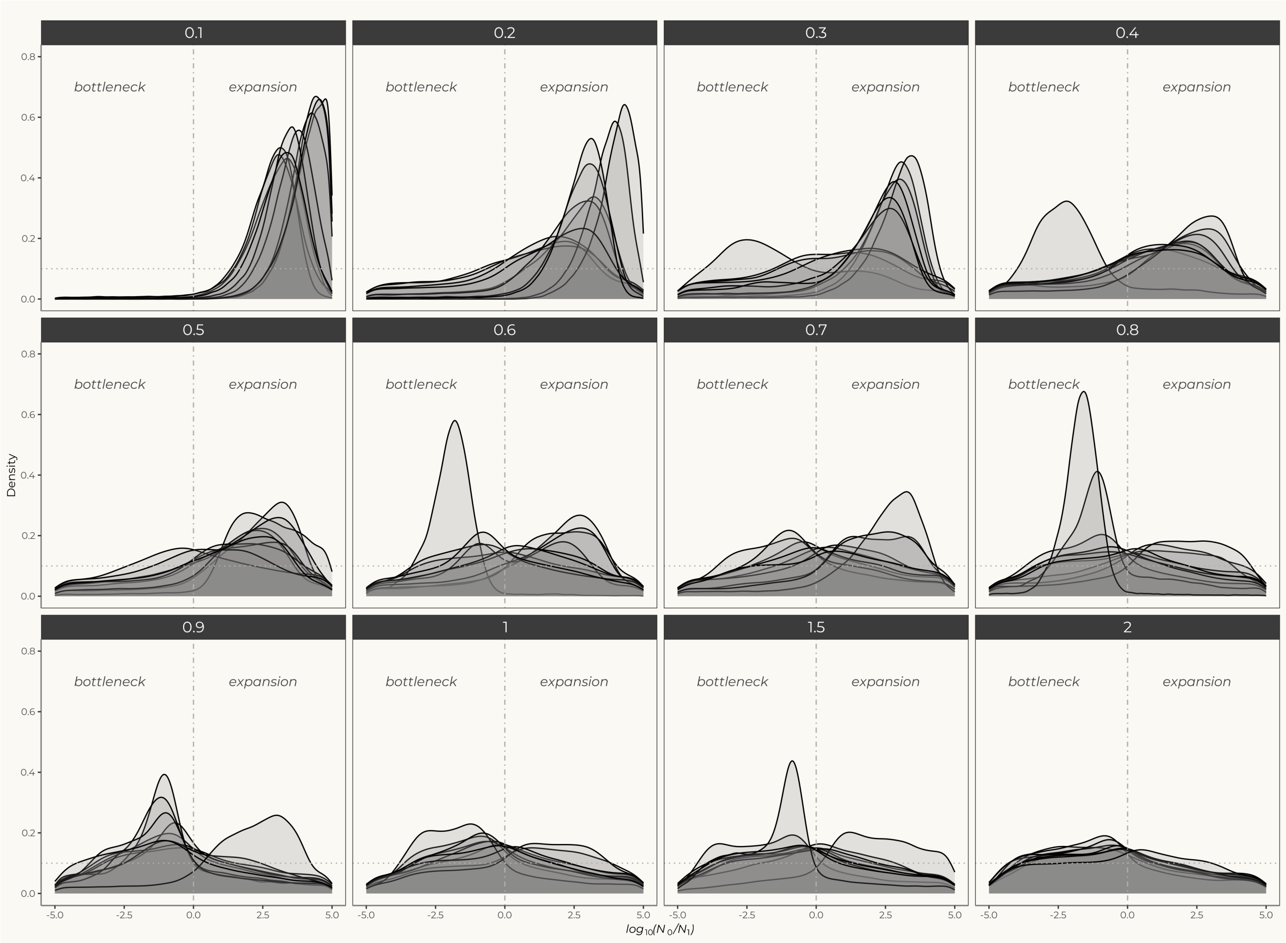

Figure S6: Posterior density for log_{10}(r), the log ratio of present (N_0) over past (N_1) population size inferred using msvar software. Each panel represents a different value of θ, varying from 0.1 to 2, and also equal to 1.5 and 2. Estimates from datasets obtained under ms. For corresponding diversity values see S7.

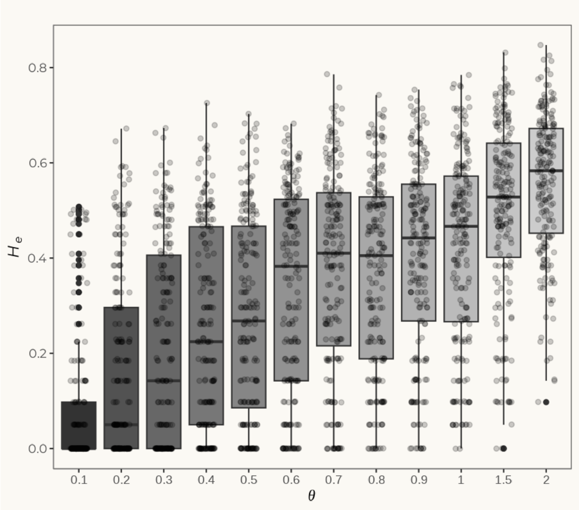

Figure S7: Expected heterozygosity, H_e for the different datasets obtained in *ms* under different θ values. Each dot is H_e estimated for one locus, according to Nei, 1978.

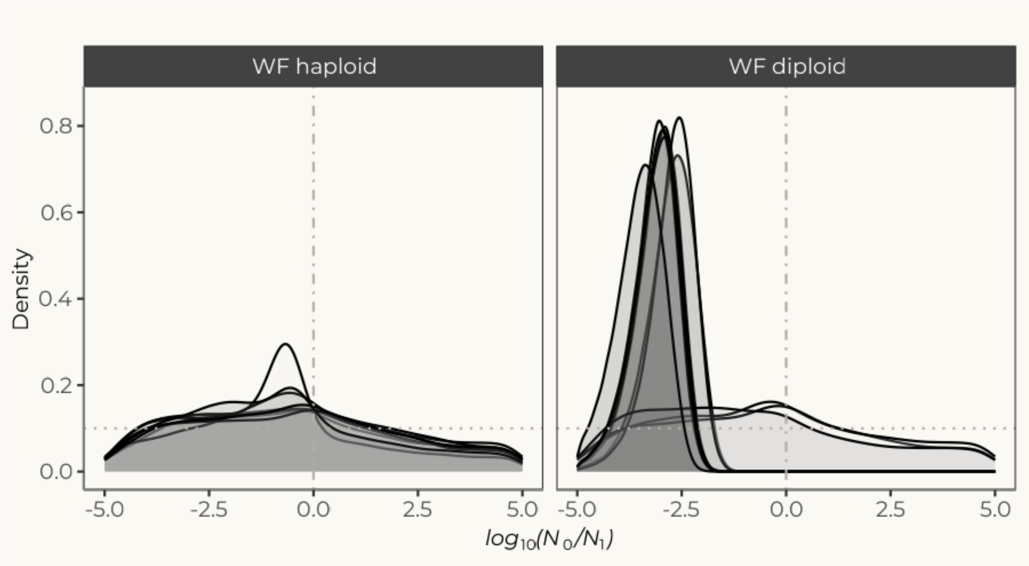

Figure S8: Posterior density for log_{10}(r), the log ratio of present (N_0) over past (N_1) population size inferred using msvar software. Panels represent Wright-Fisher scenarios of 2000 haploids and 1000 diploids (500 ♂s and 500 ♀s), respectively. Data generated under the EASYPOP program.

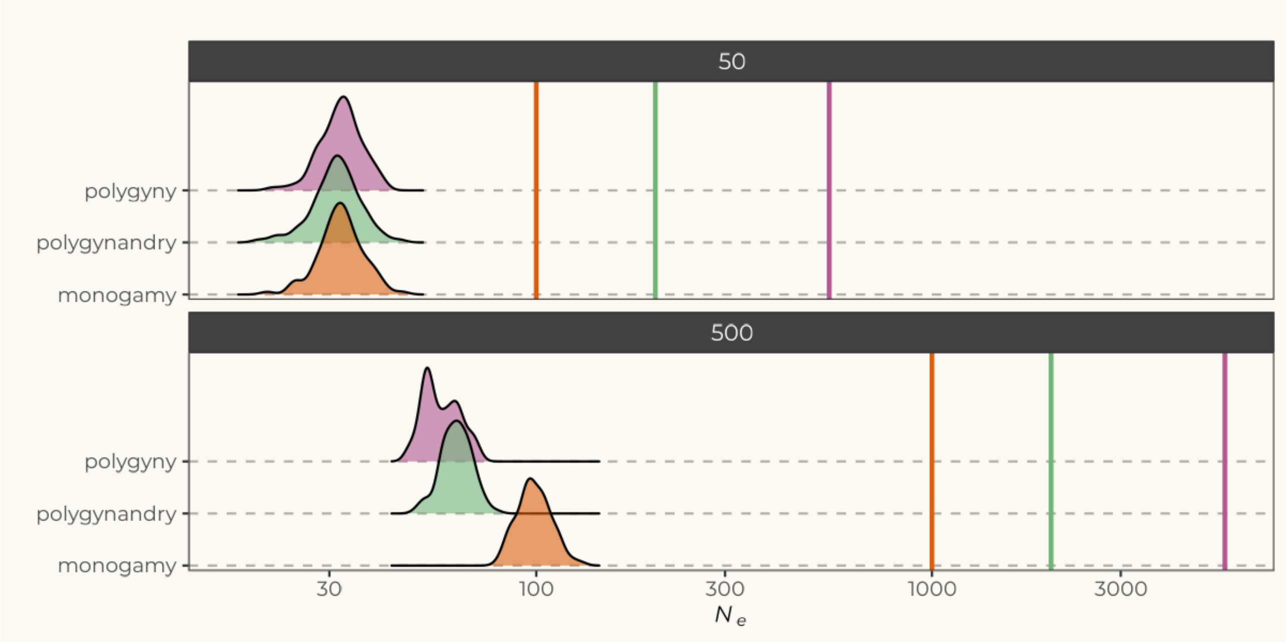

Figure S9: The distributions of N_e estimates obtained with 50 and 500 social groups. 100 individuals were sampled at random. Vertical lines are the total number of RS individuals in each scenario: $n_{RS} =$ 100, 200, 550 in 50 social groups and $n_{RS} =$ 1000, 2000, 5,500 in 500 social groups. The number of RS is used as a comparison simply because the number of breeding individuals is often considered relevant for conservation. But we note that here the number of RS is not, and should not be, interpreted as a proxy for N_e.

References

Nei, M. (1978). Estimation of average heterozygosity and genetic distance from a small number of individuals. *Genetics*, *89*(3), 583–590. https://doi.org/10.1093/genetics/89.3.583
